# Reliability, validity, and responsiveness of three seated trunk control assessments used during inpatient spinal cord injury rehabilitation

**DOI:** 10.1038/s41393-026-01272-4

**Published:** 2026-09-25

**Authors:** Anne E. Palermo, Sophie Denis, Edward J. Gorgon, Rachel J. Spillane, Lynn V. Blecher, Fernanda Di Natal, Claire L. Boswell-Ruys

**Affiliations:** 1 Neuroscience Research Australia, Sydney, Australia; 2 University of New South Wales, Sydney, Australia; 3 University of Miami, Miami, USA; 4 Kolling Insitute and School of Medical Sciences, The University of Sydney, Sydney, Australia; 5 Prince of Wales Hospital, Sydney, Australia; 6 Department of Physical Therapy, College of Allied Medical Professions, University of the Philippines Manila, Manila, Philippines

**Keywords:** Health care, Spinal cord diseases, Neurological manifestations

## Abstract

**Study design:**

Cohort study.

**Objectives:**

To investigate the reliability, validity, and responsiveness of the Trunk Control Test (TCT) and two Function in Sitting Tests (FIST) for people with a spinal cord injury (SCI; FIST-SCI and Modified-FIST (mFIST)) during inpatient rehabilitation.

**Setting:**

Inpatient Rehabilitation Hospital in Sydney, Australia.

**Methods:**

Thirty adults with recent (SCI) completed four assessments led by three raters within two weeks to determine inter-rater and intra-rater reliability. Validity was determined through known-groups and Spearman Correlation analyses with the Berg Balance Scale (BBS), Spinal Cord Independence Measure, Maximal Balance Range Test, and SCI-Falls Concern Scale. Six-week follow-up responsiveness assessments and global rating of change scale (GRCS) scores were completed.

**Results:**

Neurological level of injury ranged from C4 to L2 (80% motor-incomplete SCI). Time since injury was 84 days (95% CI:64,105). The TCT and FIST-SCI had excellent reliability (ICC > 0.9). The mFIST had excellent intra-rater (ICC = 0.908) and good inter-rater reliability (ICC = 0.867). All three tests differentiated between participants who were independent and required assistance with transfers (p < 0.05). All three tests correlated with the BBS (ρ_TCT_ = 0.627, ρ_SCI-FIST_ = 0.785, ρ_mFIST_ = 0.823) (p < 0.05). Median (IQR) change scores were 2 (0, 4) (TCT), 3 (0, 6) (FIST-SCI), and 3 (0,11) (mFIST). Participants reported a GRCS improvement (45%), no change (50%), or a decline (5%) in their seated trunk control during usual care. Attrition limited responsiveness analyses.

**Conclusions:**

The TCT, FIST-SCI, and mFIST are reliable and valid seated trunk control tests in SCI early inpatient rehabilitation. Future studies should investigate responsiveness in people with motor-complete SCI.

## Introduction

Seated trunk control, or the ability to maintain balance in sitting, is altered after a spinal cord injury (SCI). Individuals with cervical, thoracic, and lumbar SCI can experience impaired seated trunk control because muscles important for trunk stability are innervated throughout the spinal cord [1]. People with tetraplegia and paraplegia rate seated trunk control as a high priority for quality of life, likely because it is related to the completion of activities of daily living like dressing, wheelchair mobility, transfers, and ambulation [2–6]. Quantifying seated trunk control is critical to clinical and research practice because it impacts many areas of life post-SCI.

Psychometrically sound outcome measures allow clinicians to measure, track change, predict future function, and identify risk of falls [5, 7]. The identification of fall risk is of high priority because 69% of wheelchair users with SCI fall at least once per year, and this number is likely to increase with the increasing age of individuals with SCI [8]. Further, the Australian and New Zealand Clinical Practice Guidelines for the physiotherapy management of people with SCI report a strong consensus recommendation to train seated trunk control [9]. A gold standard assessment to track progress of seated trunk control has not been identified [10, 11]. Further, the American Physical Therapy Association (APTA) SCI EDGE (Evaluation Database to Guide Effectiveness) task force does not recommend a measure of seated trunk control [12]. A systematic review [13] recommended the use of the Trunk Control Test (TCT) [14] for the acute setting and Function in Sitting Test for people with chronic SCI (FIST-SCI) [4] and these assessments are listed on the Spinal Cord Injury Research Evidence database. Both tests demonstrated excellent reliability and moderate to strong validity in initial psychometric analyses, including content validity following expert review [4, 14–17]. However, the average time since injury of participants was 6.7 months in the TCT development study [14] and 14 years in the FIST-SCI development study [4]. Time since injury impacts seated trunk control making it a priority to ensure assessments function well at different time points post-SCI [14, 18]. Findings from the development studies of the TCT [14, 17, 19] and FIST-SCI [4, 15] may not be translatable to early inpatient SCI rehabilitation settings because people with chronic SCI were included in the sample. The greater stability in function of someone with chronic SCI could artificially inflate the reliability of repeated measures when they are included in a sample with people with more recent SCI.

Robust measures are required to adequately assess seated trunk control in the SCI rehabilitation setting [20]. Current assessments may be limited in their ability to capture global change in seated trunk control as these often measure one aspect of balance (static, dynamic, or reactive) or movement in a single direction (e.g. modified functional reach) [10, 11]. The primary aim of this study was to explore the reliability of the TCT, FIST-SCI, and a separate modified version of the FIST (mFIST) [21] in people receiving their initial SCI rehabilitation. The secondary aims were to establish validity and investigate the responsiveness of the three tests. A clinically appropriate assessment of seated trunk control will fill the current gap in evidence-based care.

## Methods

This study was approved by South-East Sydney Local Health District ethics board (2019/ETH00638), registered on the Australian and New Zealand Clinical Trials Registry (ACTRN12623000171617), and was carried out in accordance with the Declaration of Helsinki (2013). The lead spinal physiotherapist screened all inpatients with SCI at Prince of Wales Hospital, Australia from May 2023 to July 2024. Eligible patients (Table 1) were provided with an information and consent form before pursuing informed consent.

**Table 1:** Study Eligibility Criteria.

Inclusion Criteria	Exclusion Criteria
• At least 18 years of age • Able to provide informed consent • Traumatic or non-traumatic SCI of greater than 2 weeks duration • Medically cleared to sit out of bed • Primary wheelchair user • Level and completeness of injury as follows: ○ any motor incomplete (AIS C and D) SCI between levels cervical one (C1) and lumbar two (L2) OR ○ any motor complete (AIS A and B) SCI between levels cervical six (C6) and lumbar two (L2) according to the ISNCSCI	•Diagnosis of primary neurological injury other than SCI •Untreated severe spasticity •Current pressure injury in trunk, pelvic area, or hips •Unable to properly comprehend the protocol or study procedures •Any medical conditions that prevent sitting for testing procedures •Inability to achieve a short-sitting position •Unstable angina or orthostatic hypotension •Any other medical or cognitive issues that the investigators deem to significantly impact the participant’s balance

*SCI* spinal cord injury, *AIS* american spinal injuries association (ASIA) impairment score, *ISNCSCI* international standards for neurological classification of spinal cord injury.

**Fig. 1:**
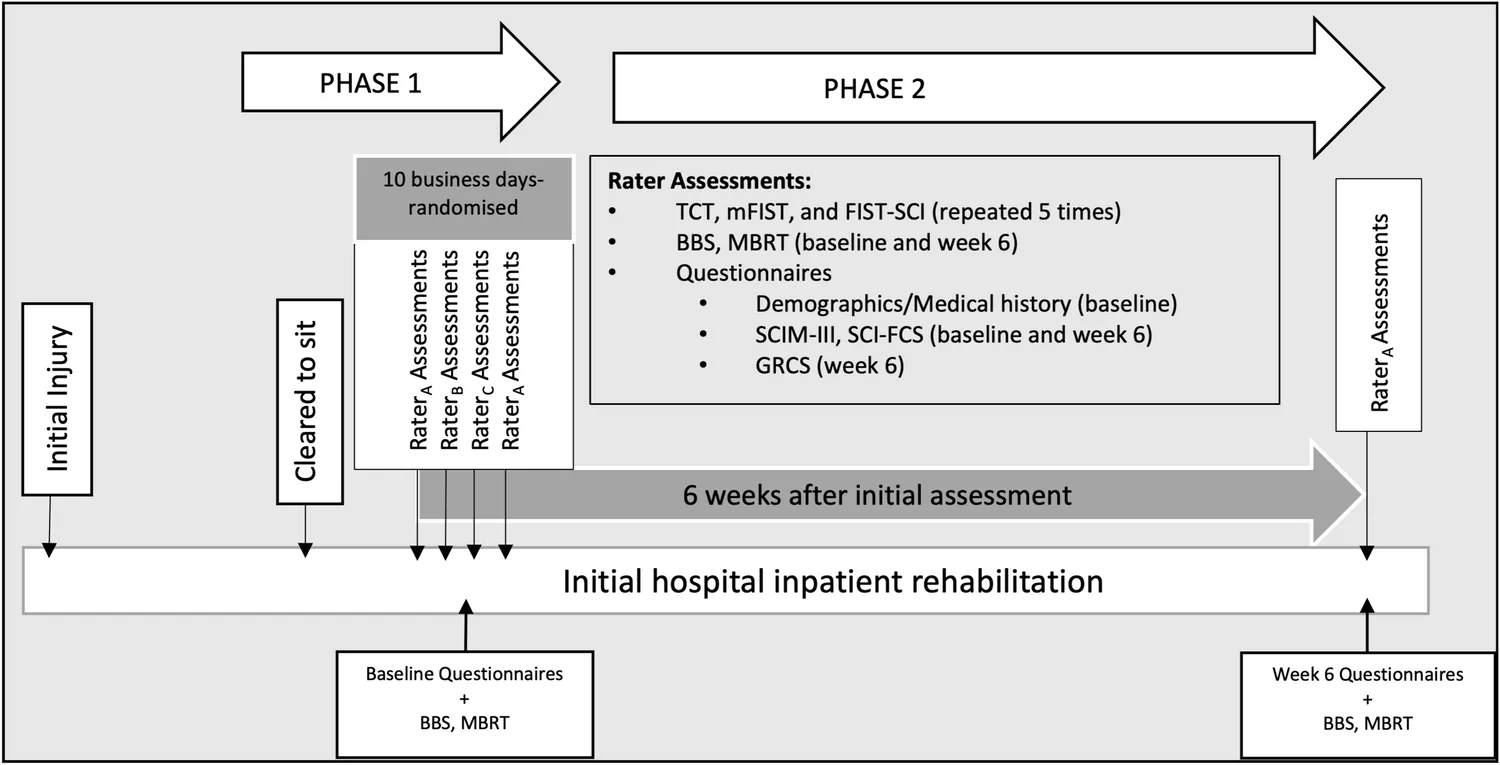
Study timeline. TCT: Trunk Control Test. mFIST: Modified Function in Sitting Test. FIST-SCI: Function in Sitting Test for people with spinal cord injury. BBS: Berg Balance Scale. MBRT: Maximal Balance Range Test. SCIM-III: Spinal Cord Independence Measure version III. SCI-FCS: Spinal Cord Injury Falls Concern Scale. GRCS: Global Rating of Change Scale.

The study consisted of two phases (Fig. 1). Phase 1 data were used to assess reliability and validity, and Phase 2 data captured responsiveness. In Phase 1, four sessions of the seated trunk control assessments (TCT, mFIST, and FIST-SCI; see Supplementary file for score sheet) were completed by three assessors (Raters A, B, and C). Rater and session order was determined by simple randomisation. The three tests were measured within the same session by each assessor, on separate days. Rater A completed two sessions for intra-rater reliability. Rater B also completed one instance of the Berg Balance Scale (BBS). A separate rater (Rater D) completed the Maximal Balance Range test (MBRT), Spinal Cord Independence Measure-III (SCIM), Spinal Cord Injury Falls Concern Scale (SCI-FCS) and recorded demographic information and medical history. If Rater D was unavailable these assessments were performed by Rater C. All Phase 1 data were collected within 10 business days. All past assessment scores were kept hidden from each assessor, and themselves if acting as Rater A.

Phase 2 was initiated six weeks after the initial assessment session. In Phase 2, Rater A re-assessed the three seated trunk control assessments. Rater B completed the BBS. Rater C or D completed the MBRT, SCIM, SCI-FCS, and a global rating of change scale (GRCS).

Assessments Completed:

*Seated Trunk Control Assessments:* The TCT [14], mFIST [21] and FIST-SCI [4] are observational outcomes. The TCT is graded based on assistance provided by the upper limbs of the participant. The mFIST and FIST-SCI are graded based on the amount of external assistance provided by the assessor. The TCT has 13 items (total score range 0–24). The mFIST and FIST-SCI have 14 items (total score range 0–56). A minor alteration was made to item 8 on the FIST-SCI. Instead of turning to touch a piece of tape, which is difficult to feel, participants could touch a bottle or pen/marker, to assess seated trunk control and not sensation. This minor alteration may limit the comparability of the FIST-SCI with previous versions where only tape was used. The inter-rater minimal detectable change (MDC) score of the FIST-SCI is 4 points [4] and TCT is 1.13 points [16], but is not yet established for the mFIST. All assessments were completed in a private room within the hospital on a height-adjustable plinth, with most tasks performed in the seated position. (Supplementary Material A for more information).

*Berg Balance Scale (BBS):* The BBS is a measure recommended for use in people with SCI by the APTA SCI EDGE Task Force [12]. The BBS has 14 items and scores range from 0–56, however only 1 item directly addresses seated trunk control.

*Maximal Balance Range Test (MBRT):* The MBRT, validated in people with SCI [18], uses a sway-meter to measure the unsupported maximal forward and backward seated leaning distance (mm). This distance is corrected for trunk height measured from the surface of the plinth to the centre of the sway-meter strap (full test range (mm) * mean trunk height of sample (cm)/trunk height of participant (cm)).

*Questionnaires:* The SCIM-III quantifies independence levels on three subscales: activities of daily living (SCIM-III_ADL_), respiratory and sphincter management, and mobility (SCIM-III_Mobility_) [22]. The SCI-FCS [23] is a self-reported questionnaire that scores concern about falling during 14 activities. Each activity is scored between 1 (not at all concerned) and 4 (very concerned), for a total score out of 56 [23]. The GRCS allowed participants to rate a change in their “sitting ability” over the last 6 weeks on a 15-point scale from -7: “A very great deal worse” to +7: “A very great deal better”. A 5-point change on the GRCS is considered important [24].

Assessors:

All assessors were experienced neurological physiotherapists. Eight assessors were trained to conduct the seated trunk control assessments through in-person and video-review sessions. After practicing on each other while acting out various trunk impairments, the raters practiced the assessments on the first eligible person. This participant consented to participate in the study, but their data were not analysed. Assessors compared their ratings with each other and discussed differences. This level of training is likely reproducible in the clinical setting. Authors who developed the TCT, mFIST, and FIST-SCI provided clarification about assessment and grading procedures as needed. Seven assessors completed at least one combined seated trunk control assessment. Assessors were chosen to perform assessments based on availability.

Outcomes and Statistical Analysis:

Inter-rater reliability was the primary outcome. The first assessment of Rater A and the assessments of Rater B and C were used to calculate inter-rater reliability. The two Phase 1 assessments of Rater A were used to calculate intra-rater reliability. Intraclass correlation coefficients (ICC) using 2-way random effects model of single measures were calculated. The sample size calculation, based on three observations, alpha=0.05, power=90%, and an ICC ≥ 0.5, accounting for 20% dropout rate, was 30 participants [25].

Three forms of validity were assessed. First, Mann-Whitney U tests analysed known-groups validity to determine hypothesised group differences. We hypothesised there would be differences in seated trunk control based on anatomy (cervical versus thoracolumbar SCI) and function (those who transfer independently versus those who require assistance). Spearman Correlations analysed convergent and concurrent validity relationships. For concurrent validity, we hypothesised that the TCT, mFIST, and FIST-SCI would have positive moderate relationships with the BBS, SCIM-III total score and subscales, height-corrected MBRT, and neurological level of injury. We hypothesised that there would be a moderate negative relationship between the SCI-FCS, and TCT, mFIST, and FIST-SCI. For convergent validity, we hypothesised that the TCT, mFIST, and FIST-SCI would have strong positive relationships. Median scores of the three assessors in Phase 1 were used in validity analyses. Responsiveness was measured by calculating change over time (Phase 2 Rater A – Phase 1 Rater A, first assessment). The raw change scores were compared to self-reported GRCS scores and previously established MDC scores of the FIST-SCI [4] and TCT [16]. Analyses were completed in IBM SPSS Statistics (V29) and alpha was 0.05.

Last, the number of “not tested” or missing items in each measure was reported. Items were considered “not tested” if a participant declined to perform a task because of pain, fatigue, or dizziness, or if an assessor decided the task posed a safety risk to the participant. Missed or not graded items without a reason were reported as human error.

## Results

Thirty people with SCI (C2 to L2, AIS A-D) were enrolled in the study and completed Phase 1 testing. Phase 1 assessments were completed within a mean of 8 [95%CI] [7–10] days. Twenty-three people completed some portion of Phase 2 data collection. Phase 2 assessments were completed a mean of 43 [41–45] days after Phase 1. If a participant’s hospital discharge date was moved forward, Phase 2 assessments were attempted if at least four weeks had passed since the first assessment. Eight people were lost to follow-up due to changes in discharge dates and one person died due to non-study related medical complications before Phase 2 data collection (Table 2).

**Table 2:** Baseline descriptors and phase 1 study outcomes.

	N (%)	Mean [95% CI]	Median [IQR]
Women	8 (27%)		
Cervical SCI	15 (50%)		
Thoracic SCI	13 (43%)		
Lumbar SCI	2 (7%)		
AIS A + B	6 (20%)		
AIS C + D	24 (80%)		
Age (years)		45 [38–53]	41 [24,66]
Time Since Injury (days)		84 [64–105]	82 [48,98]
Weight (kg)		86 [79–92]	84 [73, 101]
TCT Baseline (0–24)		19 [17–20]	18 [16,22]
mFIST Baseline (0–56)		44 [39–48]	47 [37,55]
FIST-SCI Baseline (0–56)		48 [44–52]	51 [45,56]
MBRT (mm)		470 [381–559]	506 [244, 686]
MBRT-Ht		499 [399–598]	481 [447, 620]
SCIM-III		47 [39–56]	46 [26,66]
SCIM-III_ADL_		11 [9–13]	13 [4,15]
SCIM-III_Mobility_		12 [8–15]	12 [5,15]
SCI-FCS		21 [15–26]	16 [15,24]
BBS		14 [8–20]	4 [4,30]

*SCI* spinal cord Injury, *AIS* American spinal injuries association (ASIA) impairment score, *TCT* trunk control test, *mFIST* modified function in sitting test, *FIST-SCI* function in sitting test for people with SCI, *MBRT* maximal balance range test, *MBRT-Ht* MBRT corrected for participant height, *SCIM-III* spinal cord independence measure version III, *SCIM-III*_*ADL*_ SCIM-III activity of daily living subscale, *SCIM-III*_*Mobility*_ SCIM-III mobility subscale, *SCI-FCS* spinal cord injury falls concern scale, *BBS* berg balance scale, *IQR* interquartile range [25%, 75%].

ICCs and [95% CI] for intra-rater reliability were: TCT = 0.907 [0.811–0.956], mFIST=0.908 [0.792–0.958], and FIST-SCI = 0.955 [0.838–0.983]. Inter-rater ICCs were: TCT = 0.908 [0.826–0.957], mFIST=0.867 [0.758–0.935], and FIST-SCI = 0.910 [0.831–0.958].

Three forms of validity were established. Mann-Whitney U analyses on known-groups found significant differences in ranked scores on the TCT (p = 0.004), mFIST (p = 0.009), and FIST-SCI (p = 0.004) between people who transferred independently (n = 11) and those who required assistance (n = 19). The mean difference [95% CI] in the TCT, mFIST, and FIST-SCI scores between these groups was 5 [1–8], 10 [2–19], and 8 [2–14], respectively. There was no significant difference in TCT (p = 0.138), mFIST (p = 0.722), and FIST-SCI (p = 0.800) ranked scores between people with cervical SCI (n = 15) and those with thoracolumbar SCI (n = 15) (TCT_difference_: 3 [−1–7]; mFIST_difference_: 0 [−10–10] and FIST-SCI_difference_: 2 [−6–11]).

**Fig. 2:**
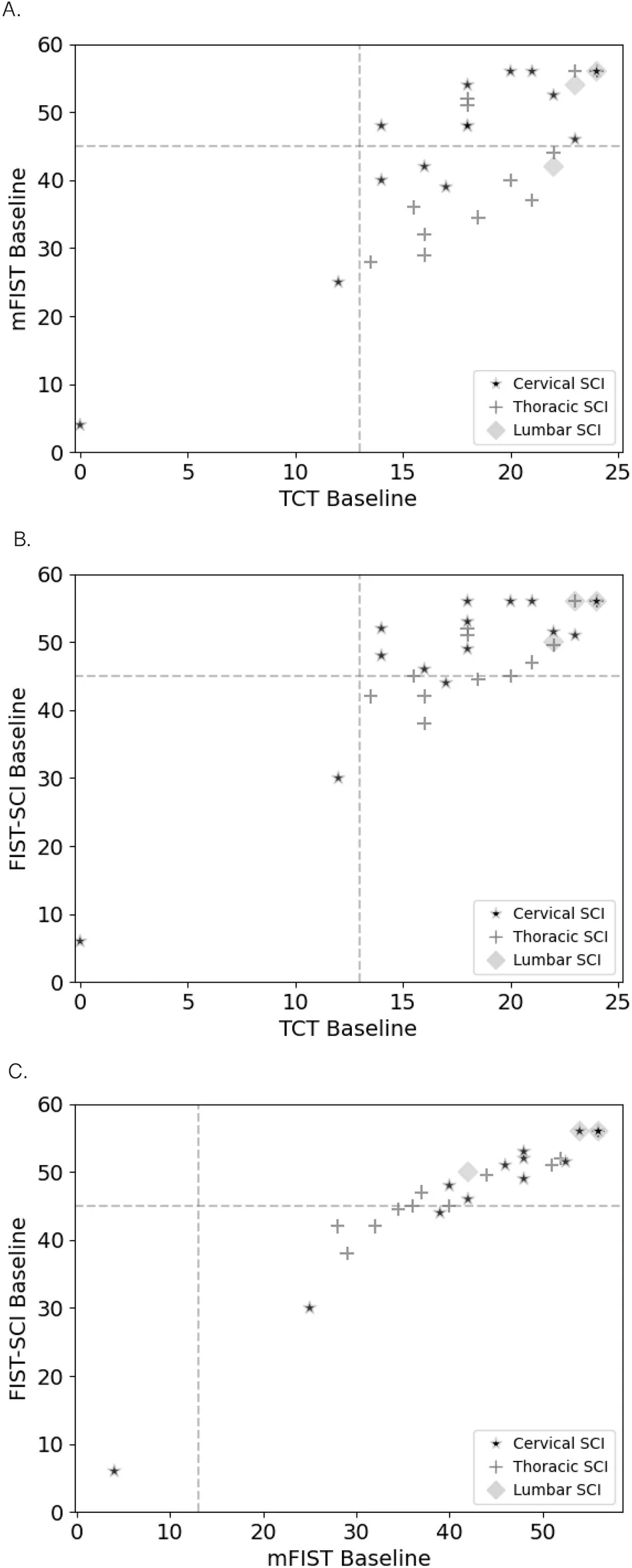
Convergent validity of Seated Trunk Control Tests. **A** mFIST v TCT, (**B**) FIST-SCI v TCT, and (**C**) FIST-SCI v mFIST. The vertical dashed line represents the cutoff score of the TCT (13 points) that discriminates those with “adequate trunk control” from those without. The horizontal dashed line represents the cutoff score of the FIST-SCI (45 points) that discriminates those with the ability to transfer independently from those who require assistance. TCT: Trunk Control Test. mFIST: Modified Function in Sitting Test. FIST-SCI: Function in Sitting Test for people with spinal cord injury.

Significant Spearman correlations were observed between the three seated trunk control tests and most variables to establish concurrent validity (Table 3). However, no significant relationship was found between the seated trunk control tests and SCI-FCS, nor between the mFIST and FIST-SCI and level of injury and SCIM-III_ADL_. The seated trunk control tests were significantly related to each other (ρ_TCT/mFIST_ = 0.741 [0.512–0.872], ρ_TCT/FIST-SCI_ = 0.725 [0.486–0.864], ρ_mFIST/FIST-SCI_ = 0.970 [0.936–0.986]) (p < 0.001), establishing concurrent validity (Fig. 2).

**Table 3:** Concurrent validity correlations.

Test	TCT	p-values	mFIST	p-values	FIST-SCI	p-values
SCIM-III	**0.586** [0.262–0.792]	**p** = **0.001**	**0.543** [0.202–0.766]	**p** = **0.003**	**0.598** [0.279–0.798]	**p** < **0.001**
SCIM-III_ADL_	**0.578** [0.257–0.784]	**p** = **0.001**	0.266 [−0.562–0.154]	p = 0.163	0.346 [−0.066–0.639]	p = 0.066
SCIM-III_Mobility_	**0.652** [0.359–0.828]	**p** < **0.001**	**0.591** [0.268–0.794]	**p** < **0.001**	**0.620** [0.311–0.811]	**p** < **0.001**
BBS	**0.625** [0.332–0.808]	**p** < **0.001**	**0.823** [0.651–0.914]	**p** < **0.001**	**0.774** [0.566–0.889]	**p** < **0.001**
MBRT-Ht	**0.596** [0.275–0.797]	**p** < **0.001**	**0.821** [0.639–0.916]	**p** < **0.001**	**0.782** [0.569–0.896]	**p** < **0.001**
NLI	**0. 462** [0.111–0.711]	**p** = **0.01**	0.114 [−0.268–0.464]	p = 0.550	0.125 [−0.256–0.473]	p = 0.509
SCI-FCS	−0.133 [−0.469–0.275]	p = 0.559	−0.236 [−0.562–0.154]	p = 0.218	−0.214 [−0.547–0.264]	p = 0.264

Correlations for the TCT Trunk Control Test, *mFIST* modified function in sitting test, and *FIST-SCI* function in sitting test for people with spinal cord injury, when compared to the total score of the SCIM-III: Spinal Cord Independence Measure version III and its subscales (*ADL* activities of daily life and mobility), the *BBS* Berg Balance Scale, the *MBRT-Ht* Maximal Balance Range Test corrected for height, *NLI* neurologic level of injury, and the *SCI-FCS* Spinal Cord Injury Falls Concern Scale. p < 0.05 was considered significant for Spearman correlations with coefficients and corresponding 95%CI presented. Significant p-values and coefficients are bolded.

**Fig. 3:**
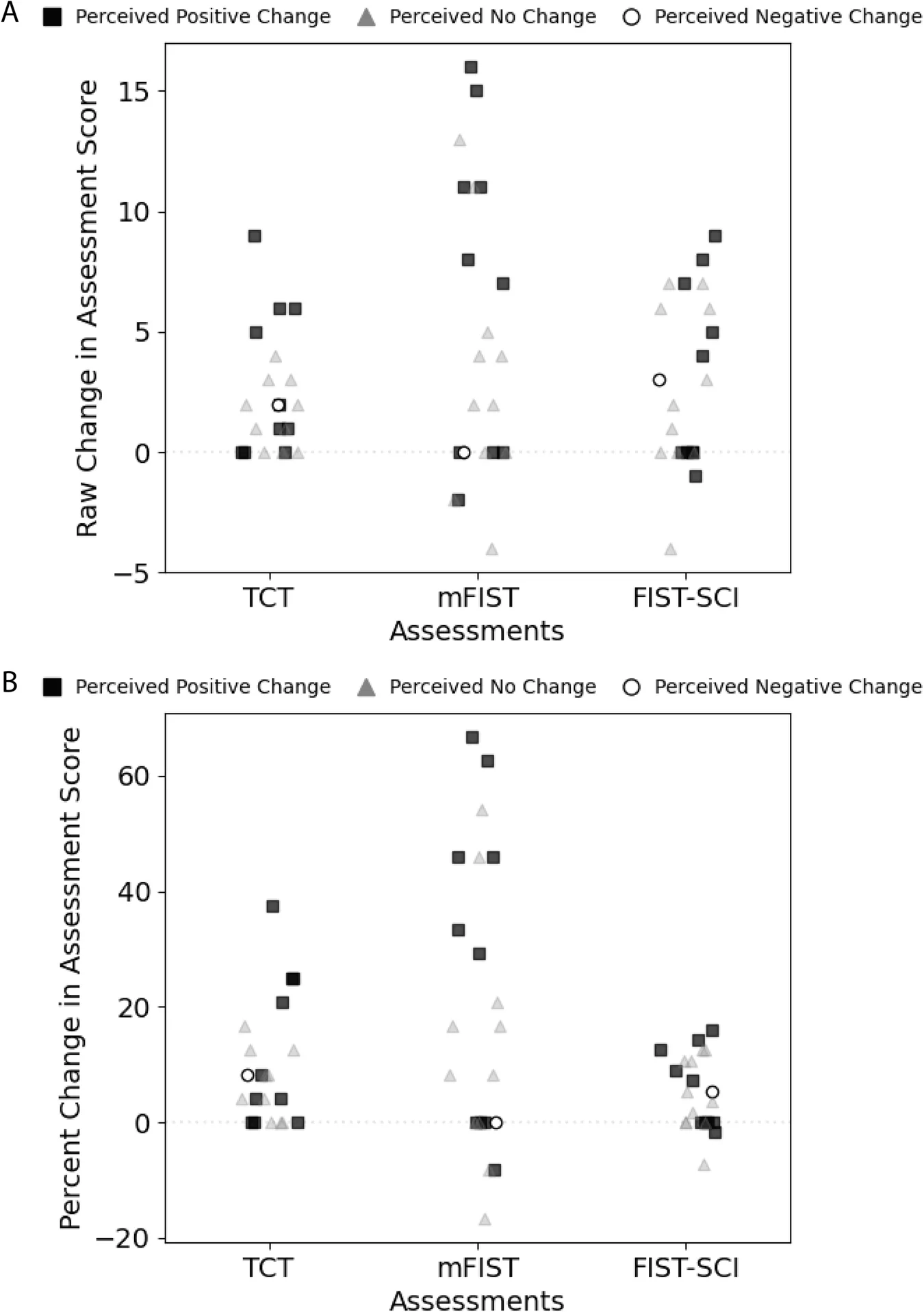
Seated Trunk Control Change Scores. Perceived positive change, perceived no change, and perceived negative change for both (**A**) Raw and (**B**) Percent changes for the TCT: Trunk Control Test, mFIST: Modified Function in Sitting Test, and FIST-SCI: Function in Sitting Test for people with spinal cord injury.

The mean [95%CI] and median (IQR) change scores on the TCT (2 [1–3], 2 (0, 4)), mFIST (5 [2–7], 3 (0,11)), and FIST-SCI (3 [1–4], 3 (0, 6)) were calculated with other study measures (Table 4, Fig. 3). No statistical analyses were completed on the change scores because missing follow-up data would have put the analyses at risk of low power. However, 95% confidence intervals were calculated and included the zero value for the MBRT and SCI-FCS. Ten of the 22 people with GRCS data reported improvements in their seated trunk control (GRCS ≥ + 5) while one person reported a decline (−7). Individual data were compared to the previously established FIST-SCI MDC of 4 [4] and TCT of 1.13 (rounded to 2) [16]. Nine of the 22 participants with Phase 2 FIST-SCI data had a positive change in score of at least 4, and five of those nine participants reported a GRCS ≥ + 5. The FIST-SCI score of one person decreased by 4 but it was not the same person who reported a GRCS of −7. Eleven of the 21 participants with Phase 2 TCT data had a positive change of at least 2. Five of those participants also reported a GRCS ≥ + 5 and one reported a GRCS of −7.

**Table 4:** Phase 2 means and change scores.

Measure	N	Phase 2 Mean [95%CI]	Mean Change [95%CI]	Median Change (IQR)
TCT	21	21 [20–23]	2 [1–3]	2 (0, 4)
mFIST	22	48 [44–52]	5 [2–7]	3 (0, 11)
FIST-SCI	22	51 [49–53]	3 [1–4]	3 (0, 6)
SCIM-III	17	60 [48–71]	14 [7–21]	13 (5, 19)
SCIM-III_ADL_	20	13 [11–16]	2 [1–4]	2 (1, 4)
SCIM-III_Mobility_	18	18 [13–23]	6 [3–10]	5 (2, 8)
SCI-FCS	21	21 [16–25]	0 [−3–3]	0 (−3, 4)
BBS	23	19 [11–27]	8 [4–12]	4 (0, 16)
MBRT (mm)	17	499 [399–598]	36 [−13–87]	20 (−10, 51)
MBRT-Ht	17	524 [418–630]	61[−1–123]	31 (−5, 111)

Phase 2 means and change scores for the Seated Trunk Control measures and other study outcomes. Errors in filling out assessments or missing data caused the varied number of participants included in each change score calculation. N: number of participants with change score. TCT: Trunk Control Test. mFIST: modified Function in Sitting Test. FIST-SCI: Function in Sitting Test for people with spinal cord injury. SCIM-III: Spinal Cord Independence Measure version III. SCIM-III_ADL_: SCIM-III activity of daily living subscale. SCIM-III_Mobility_: SCIM-III mobility subscale. SCI-FCS: Spinal Cord Injury Falls Concern Scale. BBS: Berg Balance Scale. MBRT: Maximal Balance Range Test. MBRT-Ht: height corrected MBRT IQR; Interquartile range (25, 75%).

Supplementary Material A shows the assessor-reported reasons for missed or not tested items in each test. The total number of items for the TCT (126) was 4.8 times greater than those of the mFIST (22) and FIST-SCI (26). Range of motion, pain, and human error were the most common causes of missing or not tested items.

## Discussion

This study was the first to examine the reliability, validity, and initial responsiveness of the TCT, mFIST, and FIST-SCI in the acute SCI rehabilitation setting. All three assessments were found to be reliable and valid. The tests likely quantify similar constructs of seated trunk control with good positive relationships, therefore any of the three seated trunk control tests would be appropriate for inpatient rehabilitation.

The FIST-SCI and the TCT demonstrated excellent reliability in this cohort with 95% CIs spanning the “good” to “excellent” range [26, 27]. The mFIST had “good” inter-rater and “excellent” intra-rater reliability. The wide 95% CIs may show imprecision, but the lower end of all ICC confidence intervals are greater than 0.7, supporting the recommendation for their use [27]. This is the second study to find that the mFIST has “good” reliability [21]. Both the TCT and FIST-SCI allow for three attempts of each item, while the mFIST only allows for one, this may lead to its comparably greater variability in scoring. Previous FIST-SCI ICCs were similar in people with chronic SCI [4, 15]. High reliability of the TCT was found via weighted kappa in people with a mean time since SCI of 6.7 months [14]. Previous reliability assessments were conducted by two raters, this study strengthens past evidence by adding a third rater. There is potential that our cohort was more prone to rapid functional change due to the recency of injury compared to previous studies. During inpatient rehabilitation functional change is expected within two weeks. Therefore, the most reliable seated trunk control measures should be used as soon as feasible to determine a baseline level of function and gauge response to therapy.

The strong and significant relationships [28] among the seated trunk control tests provide evidence of convergent validity, that the assessments are measuring similar aspects of seated trunk control. Our concurrent validity results show strong positive relationships between both the mFIST and FIST-SCI with the BBS and MBRT [27]. All other significant relationships are considered moderate (95% CIs extending from “weak” to “strong”). Our results add to previous concurrent and convergent validity assessments, which found the TCT to be strongly related to the SCIM [29], upper extremity motor score, and motor score of the Functional Independence Measure [17]. The TCT is moderately related to the Trunk Assessment Scale for Spinal Cord Injury, lower extremity motor score, walking index for spinal cord injury [17], and time since injury [14]. The FIST-SCI was moderately related to item three of the Motor Assessment Scale [30] (a measure of seated trunk control) and level of injury [4]. Our known-groups validity results for the TCT and FIST-SCI echo previous results, in the ability to discriminate individuals who can transfer independently and those who require assistance [4]. However, the FIST-SCI previously found differences between individuals with cervical, upper thoracic, and lower thoracic SCI [4], but our current seated trunk control scores did not differ significantly between people with cervical and thoracolumbar injuries. This lack of difference in our current study is likely due to the high proportion of people with motor-incomplete SCI in our sample (80%), compared to the FIST-SCI validity study (30%; unpublished data) [4]. In addition to known-groups validity, our results establish strong convergent validity between the seated trunk control tests, strong concurrent validity for the mFIST and FIST-SCI and moderate concurrent validity for the TCT in the acute SCI rehabilitation setting. Relationships for the tests with confidence intervals spanning from “weak” to “strong” should be investigated in a larger, more heterogenous sample [28].

The time of recruitment of the sample of convenience could have also impacted the lack of variability in our sample outcomes, which may have led to our non-significant correlations. About 50% of the seated trunk control change scores did not reach an MDC. The SCI-FCS scores were also lower at baseline than previous studies [20, 23] and largely unchanged after 6 weeks. We may have recruited some individuals who had already plateaued in their seated trunk control, which could limit the variability in the sample. Relationships between seated trunk control and other factors, like concern about falling and level of injury, may become less detectable after a plateau is reached.

This is the first study to report seated trunk control change scores for a sample of people with both tetraplegia and paraplegia in early inpatient SCI rehabilitation. Paglierani et al. [31] reported the TCT improved an average of 7.8 points from admission (average 2.7 months post-SCI) to discharge in people with paraplegia [31]. The mean change in our sample was less (2 [1,3]). The mean TCT score of our sample at baseline was 19 [17,20] while the Paglierani study was 11.4 (SD: 5.7) [31]. The high Phase 1 scores could have caused a ceiling effect in our study. Additionally, Paglierani et al used a longer follow-up period (average 3.3 months) which could have allowed for greater functional recovery. Harvey et al [20] reported change scores for the MBRT (maximal lean test) in a control group that received 6 weeks of usual care as part of a randomised controlled trial. The group (recruited on average 11 weeks post-SCI) improved by an average of 81 mm (65%) in the MBRT [20]. Our study found an average change of 36 mm (8%) on the MBRT. The initial mean MBRT of the Harvey et al. control group was 125 mm, signalling that our sample, with a mean Phase 1 MBRT of 470 mm, was already higher functioning. The average percent changes in the MBRT and other seated trunk control tests reported by Harvey et al are greater than those in the current study and may not quantify all the aspects of seated trunk control that are captured in the FIST-SCI or TCT. The differences are likely also impacted by the dissimilarities in the two samples. All 16 participants in the control group in Harvey et al trial had motor-complete paraplegia, while 80% of our sample had motor-incomplete SCI. People with motor-incomplete SCI would likely perform better at baseline and may reach a ceiling effect in some of the reported outcomes compared to those who had a motor-complete SCI.

The three seated trunk control measures are valid and reliable. We completed the assessments in one timed session but did not time individual assessments. It is reported that the average time to complete the TCT is 8 min [14] and 13 min for the FIST-SCI [4]. Time data is not yet available for the mFIST, yet would be similar to the FIST-SCI. Additionally, there is no specialised equipment required beyond what can be found in a neurological rehabilitation setting. However, there may be limitations inherent to the inpatient rehabilitation setting that impact their ease of use. Pain, range of motion limitations, and human error were reported as reasons for not completing items on the TCT at a higher frequency than the mFIST and FIST-SCI. For example, many participants were unable to maintain the cross-legged position required for the TCT due to hip pain or range of motion limitations. In addition, participants with neck braces were unable to complete the third item of the mFIST and FIST-SCI that asks them to turn their neck. Improvements in pain, hip range or the absence of braces in Phase 2 may impact the change scores or responsiveness of the tests. Higher scores should be interpreted as improved ability to complete any functional assessment-related task, not necessarily the return of neurological function.

## Limitations

Findings need to be interpreted with consideration of the study limitations. Due to missing data, we did not meet the a priori power analysis sample size of 24 participants in the inter-rater reliability analyses. While the mFIST reached this sample size, data from 22 and 23 individuals were included in inter-rater reliability analyses for the TCT and FIST-SCI, respectively. The observed power may be less than 0.90, however, the 95% CIs for the inter-rater ICC fall above 0.8 or can be interpreted as “good” and the TCT and FIST-SCI can be recommended for use [27]. Further, our highly performing and motor-incomplete cohort may have experienced ceiling effects. In Phase 1, only two participants scored below a previously established TCT cut-off of 13 that determines “adequate trunk control” [14] while seven fell below the FIST-SCI cut-off score of 45 that determines whether assistance is required for transfers (Fig. 2) [4]. This predominantly incomplete sample may limit generalizability to other samples or patients that have differing proportions of AIS scores like those in the original TCT and FIST-SCI samples in which <50% of participants had motor-incomplete SCI [4, 14]. Responsiveness analyses to identify minimal clinical important differences for each test could not be performed due to 6-week attrition. Due to the limited Phase 2 data collected in this study, future studies within SCI rehabilitation units should capture TCT and FIST-SCI scores on admission and discharge and GRCS scores to capture a more complete picture of normative values and the responsiveness of these measures. Last, developers of the seated trunk control assessments were either contacted to prepare for assessor training (TCT, mFIST), or were directly involved in study development, assessor training, assessment, and analysis (FIST-SCI, author: AP). To limit bias of the developers, the study was registered and the analysis plan was generated with a statistician prior to the start of data collection.

## Conclusion

The mFIST shows similar validity to the TCT and FIST-SCI, however, the TCT and FIST-SCI have excellent reliability and may be more appropriate for research and clinical use during inpatient rehabilitation. There may be clinical barriers, including pain and limited range of motion, to the use of all three tests. The preliminary report of responsiveness in this study may have been limited by the higher-functioning cohort comprised primarily of participants with motor-incomplete SCI and the limited Phase 2 completion. The ability of the seated trunk control tests to capture change should be assessed further, in a sample of individuals with motor-complete SCI. The seated trunk control tests could be used to guide the assessment and treatment of trunk impairment in early inpatient SCI rehabilitation.

## Supplementary information

Supplemental A

Supplemental B

## Data Availability

Data are available from the corresponding author on reasonable request.
