# Supplementary material for: Reliability, validity, and responsiveness of three seated trunk control assessments used during inpatient spinal cord injury rehabilitation
Source: Spinal Cord. 2026 Sep 25;64(11):1011–8. doi: 10.1038/s41393-026-01272-4 (PMC13731139; doi:10.1038/s41393-026-01272-4)
Supplement: Supplementary file 1 [file 41393_2026_1272_MOESM1_ESM.docx]

|  | **TCT** | | | | **Modified-FIST** | | | | **FIST-SCI** | | | |
| --- | --- | --- | --- | --- | --- | --- | --- | --- | --- | --- | --- | --- |
| *Missing/Not Completed Items* | TCT Total Missing Items: 126 | | | | Modified-FIST Total Missing Items: 22 | | | | FIST-SCI Total Missing Items: 26 | | | |
|  | **Phase 1 Task** | **Missing** | **Phase 2 Task** | **Missing** | **Phase 1 Task** | **Missing** | **Phase 2 task** | **Missing** | **Phase 1 Task** | **Missing** | **Phase 2 Task** | **Missing** |
|  | Crossleg Left | 20 | Crossleg Left | 3 | Scooting | 5 | Touch feet/floor | 2 | Scooting | 8 | Touch feet/floor | 2 |
|  | Upright to Supine | 19 | Crossleg Right | 3 | Posterior Nudge | 4 | Forward Lean | 1 | Touch feet/floor | 5 |  |  |
|  | Roll Right | 17 | Touch feet/floor | 3 | Forward Lean | 3 |  |  | Posterior Nudge | 4 |  |  |
|  | Roll Left | 13 | Roll Right | 3 | Lateral Nudge | 2 |  |  | Lateral Left Nudge | 2 |  |  |
|  | Crossleg Right | 14 | Roll Left | 3 | Anterior Nudge | 2 |  |  | Lateral Right Nudge | 1 |  |  |
|  | Touch feet/floor | 9 | Upright to Supine | 2 | Posterior Reach | 1 |  |  | Head Rotation | 1 |  |  |
|  | Forward lean Left | 5 |  |  | Head Rotation | 1 |  |  | Tough Posterior | 1 |  |  |
|  | Lateral Lean Right | 5 |  |  | Touch feet/floor | 1 |  |  | Forward lean | 1 |  |  |
|  | Lateral Lean Left | 4 |  |  |  |  |  |  | Lateral Lean Left | 1 |  |  |
|  | Forward lean Right | 3 |  |  |  |  |  |  |  |  |  |  |
|  | *Phase 1: Missing items* | 109 | *Phase 2: Missing items* | 17 | *Phase 1: Missing items* | 19 | *Phase 2: Missing items* | 3 | *Phase 1: Missing items* | 24 | *Phase 2: Missing items* | 2 |
|  | *Phase 1: Missing items per test* | 0.84 | *Phase 2: Missing items per test* | 0.77 | *Phase 1: Missing items per test* | 0.16 | *Phase 2: Missing items per test* | 0.14 | *Phase 1: Missing items per test* | 0.2 | *Phase 2: Missing items per test* | 0.09 |
| *Reasons Reported for Missing/Not Completed Items* | **Reason Phase 1** | **Reports** | **Reason Phase 2** | **Reports** | **Reason Phase 1** | **Reports** | **Reason Phase 2** | **Reports** | **Reason Phase 1** | **Reports** | **Reason Phase 2** | **Reports** |
|  | Pain | 41 | Pain | 10 | Human error | 11 | ROM | 3 | Human error | 12 | ROM | 2 |
|  | ROM | 22 | Dizziness | 2 | Request to stop | 3 |  |  | Pain | 6 |  |  |
|  | Human Error | 21 | ROM | 2 | Neck collar | 2 |  |  | ROM | 3 |  |  |
|  | Fear | 5 | Shoulder instability | 1 | Pain | 1 |  |  | Shoulder instability | 3 |  |  |
|  | Shoulder instability | 2 | Human error | 2 | Fear | 1 |  |  |  |  |  |  |
|  | Dizziness | 3 |  |  | ROM | 1 |  |  |  |  |  |  |
|  | Fatigue | 1 |  |  |  |  |  |  |  |  |  |  |
|  | Bowel | 1 |  |  |  |  |  |  |  |  |  |  |
|  | No reason | 4 |  |  |  |  |  |  |  |  |  |  |
|  | *Total Reports in Phase 1* | 109 | *Total Reports in Phase 2* | 17 | *Total Reports in Phase 2* | 19 | *Total Reports in Phase 1* | 3 | *Total Reports in Phase 2* | 24 | *Total Reports in Phase 2* | 2 |

**Supplementary Material A.**

Unreported or Missing Items of the Seated Trunk Control Tests. The clinical utility chart for the Trunk Control Test (TCT), Modified Function in Sitting Test (Modified-FIST) and the Function in Sitting Test for Spinal Cord injuries depicting the number and reasons why items were not completed in Phase 1 and 2 of testing. ROM: Range of Motion. The “No reason” refers to participants scoring zero on items that were not explained in the comment section and “Human error” refers to missed scoring of items.
