# Supplementary material for: Reliability, validity, and responsiveness of three seated trunk control assessments used during inpatient spinal cord injury rehabilitation
Source: Spinal Cord. 2026 Sep 25;64(11):1011–8. doi: 10.1038/s41393-026-01272-4 (PMC13731139; doi:10.1038/s41393-026-01272-4)
Supplement: Supplementary file 2 [file 41393_2026_1272_MOESM2_ESM.docx]

**Supplementary Material B.**

Seated Trunk Control Assessment Instructions and Guidance. Detailed assessment instructions were taken from publications and/or from the developers of the assessments. These tables and notes were used to train study assessors and were available during each assessment for reference. The item numbers in the instructions section for each assessment correspond to the item numbers in our scoring sheet, also found in this section.

**mFIST Directions and Setup**

- “Try to do each task by yourself and ask for help if you need it.”
- “Try to do each task to the fullest extent of your ability and come back to starting position.”
- “You may use your hands if needed for balance but try to do each task without your hands first.”
- “Use your stronger side for one-sided tasks.”

½ femur (50% thigh support) on surface. Hips & knees at 90 degrees

Randomly administer each nudge once. Use two fingers to nudge.

| **mFIST Scoring Scale** | |
| --- | --- |
| **4** | Independent |
| **3** | Verbal cues/increased time |
| **2** | Upper extremity bracing/support for balance |
| **1** | Needs assistance to complete task  *document level of assist needed (mid/mod/max) *supervision not included* |
| **0** | Dependent (unable to complete task, even with physical assist) |

| **mFIST** | |
| --- | --- |
|  | **Anterior nudge**: to superior sternum |
|  | **Posterior nudge:** between scapular spines |
|  | **Lateral nudge:** to dominant side acromion |
| **1** | **Static sitting:** 30 seconds  “Sit with your hands in your lap.”  Hands may be clasped together or palms facing upward. |
| **2** | **Sitting, eyes closed**: 30 seconds  “Close your eyes and remain sitting with your hands in your lap, palms up.” |
| **3** | **Sitting, head rotation:** left and right  “Sit upright and look forward. Now, look right and hold for 2 sec. Now, look left and hold for 2 sec. Return your head to center and look forward.” |
| **4** | **Sitting, lift thigh:** foot must clear ground 1 inch Circle leg lifted: R / L  “Lift one thigh so that your foot comes 1 inch off the ground and hold for 2 sec.”  Allowed to use upper extremities to lift leg without downgrading score. |
| **6a** | **Pick up object from behind:** object (empty water bottle) at midline, hand’s breadth posterior to sacrum  “Turn around and pick up the water bottle I’ve placed behind you.”  Seated pivot allowed without downgrading the score if hand/arm use is not available to the participant.***SEE NOTES FOR MORE ON THIS*** |
| **7a** | **Forward Reach: use dominant arm, full available ROM ***SEE NOTES FOR LACKING ARM FUNCTION*****  Demonstrate task first and perform movement passively to assess patient’s full ROM.  “*Reach with your dominant arm as far as you can while staying balanced. Keep your other hand, palm up remaining in your lap*.”  Full score includes: movement through full available ROM or when abdomen contacts anterior thighs and  come back to sitting.  **Circle arm: R / L Length acromion to wrist:_______ Length of reach:_______ Reach/Arm length: _____%** |
| **8a** | **Lateral Reach: use dominant arm, must clear opposite ischial tuberosity Circle arm used: R / L ***SEE NOTES FOR LACKING ARM FUNCTION*****  Demonstrate task first.  “Keep your feet on the floor. Lift your dominant arm out to the side at shoulder height. Reach to the side until your opposite hip lifts off the mat/plinth and come back to seated.”  Full score includes: maintaining upright upper trunk, abducted upper extremity, contralateral trunk  shortening, clearance of contralateral ischial tuberosity, and return to sitting. **Length of reach:** _____ |
| **10a** | **Pick up object from floor: between feet at 1^st^ MTP joint**  “*Pick up the water bottle from the floor and then return to sitting position.*”  ***SEE NOTES FORM MORE ON THIS RELATING TO HAND FUNCTION*** |
| **11** | **Posterior scooting**: move backwards 2 inches “Scoot backwards 2 inches.” |
| **11** | **Posterior scooting:** move backwards 2 inches “Scoot backwards 2 inches.” |
| **11** | **Posterior scooting**: move backwards 2 inches “Scoot backwards 2 inches.” |

Informal notes from original author of mFIST:

- Item 6 and 10: Our original intent for using the mFIST was for testing seated balance and/or trunk stability. Therefore, we did NOT downgrade the score if hand function/fine motor skills were the only reason the participant could not successfully retrieving the water bottle. This was true also while reaching for the water bottle from behind. We scored based on their ability to balance /or maintain trunk stability during the entire task.
- Item 6: Even if participant has very little trunk rotation, they are graded on their ability to initiate a trunk rotational movement if they do not have hand function.
  - if the participant could not do a trunk rotation to retrieve the bottle, but they could do some level of a seated pivot to reposition to reach most of that activity, this was typically scored as a 4 (if pivot could be done without use of arms) or 2 (pivot with use of UEs – typically our population fell here). They were scored a 0 if: (1) they could not trunk rotate to attempt retrieval, and (2) could not perform a pivot without one of us essentially lifting them and moving them ourselves.
- Reaching items: If a participant was able to lift their arm “close” to shoulder level, then do not downgrade the score at all. The definition of “close” was up to PT judgement. However, typically use about 45 degrees as the cutoff (i.e. as long as they could raise their arm at least 45 degrees while performing the task, no downgrading would occur). If the participant could not lift their arms, then  downgrade the score. Typically, participants that had difficulty raising their arms to 45 degrees over these items also needed assistance to fully complete the task (and were therefore scoring a 1).

Can use anti-slip pads for feet if necessary

**FIST-SCI Directions and Setup**

Up to three trials (to minimize rater anxiety and assess need of supervision for safety) of each test item are allowed (other than the nudges: x1). Verbal directions and demonstration as needed are given by the test administrator following the directions for each item.

Position as close to the following description as their range of motion allows. Person seated at the edge of the mat, plinth, or standard hospital bed, with proximal thigh (space for three to four fingers to fit between popliteal fossa and mat/plinth) in support, hips, and knees ﬂexed to 90◦, and feet were ﬂat in support. Thighs should be positioned in neutral hip abduction and adduction and neutral rotation — hands resting comfortably unless needed for balance support. Place one piece of tape 2 inches (5.1 cm) lateral to patient/participant’s left and right trochanters and 2 inches (5.1 cm) posterior to sacrum in the midline of the pelvis. Place an additional piece of tape 5 inches (12.7cm) posterior the sacrum in the midline of the pelvis.

| **FIST-SCI Scoring Scale** | |
| --- | --- |
| **4** | **Independent**- completes the task independently and successfully *without the use of upper extremities for balance purposes*  **Item 7- able to use upper extremity to assist with leg lift if hip flexor is absent/weak* |
| **3** | ***Upper Extremity Support***- *able to complete the task independently with the use of upper extremities for balance purposes* |
| **2** | ***Supervision, Contact Guard*** *(hands-on assist or cues),* ***Minimal Assistance****- able to perform the task with 75%-100% of the effort or work provided by the patient/participant, or there is a safety concern with the patient/participant completes the task without external assistance* |
| **1** | ***Moderate to Maximal Assistance****- able to perform the task with 74-25% of the effort or work provided by the patient/participant* |
| **0** | **Complete Assistance/*Dependent/Unable****- Patient/participant is unable or refuses to perform or provides less than 25% of the effort or work to complete the task* |

**Supervision- rated based on the three trials allotted. If the assistant is behind but not involved- not rated as supervision. Supervision rating comes from the lead physio performance and clinical decision making

| **FIST-SCI** | | **Function** |
| --- | --- | --- |
|  | **Anterior nudge** (light pressure × 1, at sternum)  Without warning, at any time during testing, push participant with light pressure at the superior portion of the sternum. | Reaction to environmental perturbation |
|  | **Posterior nudge** (Once, between scapular spines) Without warning, at any time during testing, push participant with light pressure between scapular spines. |  |
|  | **Lateral nudge** (Once on each side at the acromion). Without warning, at any time during testing, push participant with light pressure at the acromion.*Record lower side and score |  |
| **1** | **Static sitting:** “Sit with your hands resting comfortably for 30 seconds.” If it appears that the individual is using their upper extremities for support the tester can ask them to lift their arms up, if possible. | Survey environment |
| **2** | **Sitting, move head side to side** “Remain sitting steady and tall without using your hands unless you need them to help you balance. When instructed ‘look right,’ keep sitting straight, but turn your head to the right. Keep looking to the right until I tell you ‘look left,’ and then keep sitting straight and turn your head to the left. Keep your head to the left until I tell you, “look straight,” and then keep sitting straight but return your head to the center.” *Test administrator will have patient/ participant hold each side for 3 seconds* |  |
| **3** | **Sitting, eyes closed**: “Close your eyes and remain sitting still with your hands resting comfortably for 30 seconds.” | Interaction in poorly lit environment |
| **4** | **Sitting, lift foot** (if one side is preferred, i.e.. transfer side, lift can be scored on that side) “Sit with your arms resting comfortably and lift your foot to clear the floor.”  *No point deduction will be made for using upper extremities to lift leg due to absence/weakness of Hip Flexor- only point deduction if upper extremities are used for balance, steadying or support.* | Preparing for transfers |
| **6** | **Turn and touch a spot/item behind you**: “Turn around and touch the furthest piece of tape/item that I’ve placed behind you.” (Tape placed in midline, 5 inches (12.7cm) posterior to hips, the subject may turn to the preferred side and use either arm)  Item may be pen/marker/block/phone/bottle | Retrieving something from the backpack, survey environment |
| **7b** | **Forward lean**: “Lean forward to unweight your ischial tuberosities/ bottom/ sitting bones and return to the upright position.” | Weight shifts and unweighting |
| **8b** | **Lateral Lean**: “Lean to the side to unweight the opposite ischial tuberosity/ bottom/sitting bone and return to the upright position.”  *Repeat on both sides, score lower performance and record side.* |  |
| **10b** | **Touch dorsum of foot**: “Touch the laces of your shoe and return to the upright position”  *Demonstrate with the dorsum of your middle finger if hand function of the patient/participant is limited | Further unweighting, retrieving object from the floor if hand function allows |
| **11** | **Posterior scooting** (2”) “Move backward 2 inches (5.1cm) without using your arms, unless you need them to help you move.” | Transfer/bed mobility |
| **11** | **Anterior scooting** (2”) “Move sideways 2 inches (5.1cm) without using your arms, unless you need them to help you move” |  |
| **11** | **Lateral scooting** (2”) “Move sideways 2 inches (5.1cm) without using your arms, unless you need them to help you move”  *Repeat on both sides, score lower performance and record side.* |  |

Can use anti-slip pads for feet if necessary

**Trunk Control Test (TCT) Directions and Setup**

The subject attempts the test on three occasions. The best attempt is scored. The observer can provide biofeedback between tests. Verbal or non-verbal (a demonstration) instructions can be given.

Initial position: seated with feet on a supporting surface, knees flexed at 90°, without trunk support, hands resting on thighs.

| **Trunk Control Test (TCT)** | | | | |
| --- | --- | --- | --- | --- |
| Study # | TCT # | Item | Scoring | |
| For the **static control**, the maintenance of the seating posture in the initial position and with a crossed leg is evaluated. The patient is asked to keep seated with the hips and knees in a flexion of 90º while laying his or her hands on both thighs without any external support. If they cannot accomplish this task, they are told that they may use their upper extremities. The score is 0 if they cannot perform it and fall, 1 if they require the upper extremities to perform it and 2 if they can maintain their posture during 10 seconds without using their upper extremities. | | | | |
| **1** | **1** | Maintain initial position during 10 sec | 0: Falls  1: Need support of upper limbs  2: Maintains position for 10 sec | |
| **5** | **2,3** | Crosses one pelvic limb over the other  Left and Right  *Can hold in place with straps* | 0: Falls  1: Needs support of upper limbs to maintain position  2: Maintains position for 10 sec | |
| **Dynamic equilibrium** to carry out activities with upper limbs. From the initial position, one of the thoracic limbs is maintained with shoulder flexion at 90°, complete elbow extension, pronation of the forearm, wrist in neutral position and fingers extended. A piece of cardboard in the form of a circle 10 cm in diameter is utilized as a dartboard. *** For all reaches, do not grade the return to upright, stop grading when the target is touched.*** | | | | |
| **7c** | **1,2** | Place the dartboard mid-line at the height of glenohumeral joint 10 cm from point of the fingers and ask the individual to touch this with right hand. **Left and Right** | | 0: Not done  1: Requires support of the contralateral limb  2: Done without support |
| **8c** | **3** | Place dartboard 45° to the right of position 1 and ask individual to touch it with right hand | | 0: Not done  1: Requires support of the contralateral limb  2: Done without support |
| **9** | **4** | Same as TCT #3 but the dartboard moves 45° to the left | | 0: Not done  1: Requires support of the contralateral limb  2: Done without support |
| **9** | **5** | Same as TCT #3 with left hand | | 0: Not done  1: Requires support of the contralateral limb  2: Done without support |
| **8c** | **6** | Same as TCT #4 with left hand | | 0: Not done  1: Requires support of the contralateral limb  2: Done without support |
| For the **dynamic control**, the trunk’s active flexion and extension against gravity are evaluated in the first two tests. The patient is asked to touch his or her feet with both hands **and to return to the initial position** as well as to **lie supine and rise to a seating position**. It is recommended that they perform this without their upper extremities but if they cannot perform it, they are encouraged to use them. The test is graded 0 if a patient cannot perform the test, 1 if they perform it with the assistance of the upper extremities and 2 if they accomplish it without help. Then, the position changes in decubitus (a very uncommon term for supine, is this required or could you say: the position changes from supine) by rolling are evaluated and graded with 1 if performed and 0 if not. | | | | |
| **10c** | **1** | Touch the feet | 0: Not done  1: Requires support with an upper limb  2: Touches feet with both hands | |
| **12** | **2** | Lie down in supine decubitus position and return to initial position | 0: Not done  1: Requires air of upper limbs  2: Done without aid | |
| **13** | **3** | Roll onto right side | 0: Not done  1: Done | |
| **13** | **4** | Roll onto left side | 0: Not done  1: Done | |

Additional notes: Reaching- can use elbow or acromion

- Midline Reach- measure anterior to the elbow/acromion

-Lateral Reach - measure 45 degrees laterally from the elbow/acromion

-Cross-Body Reach - measure 45 degrees toward midline from the elbow/acromion

| Item: Full scripts are available at the end | mFIST Score | FIST-SCI Score | TCT Score |
| --- | --- | --- | --- |
| Individuals will start in a sitting position, knees/hip at 90° or as close to the position as possible.  ***Add tape targets**Determine Dominant Side***  **Date:** ___________ **Rater Initials**: ____________  **Time start**: ____________ **- end**: _____________  **ID#: ABS**_______ **Binder: Yes No**  **Initial Test: A1 B C A2**  **6wk Test-A3** | **Score first attempt** *Dominant sides remain consistent between tests* 4= Independent 3= Verbal cues/increased time 2= Upper extremity bracing/support for balance 1= Needs assistance to complete task *Document assist (mid/mod/max) 0= Dependent (unable to complete task, even with physical assist) | **Score best of three attempts** 4= Independent, no upper extremity assist 3= Self-Assist with upper extremities 2= Requires supervision, CGA, Min Assist (<25% assist) 1= Requires Mod/Max assist (25-75% assist) 0= Dependent/unable or complete physical assist (>75% assist) | **Score best of three attempts** |
| **Anterior Nudge**: **FS/MF:** sternum | 0 1 2 3 4 | 0 1 2 3 4 | NA |
| **Posterior Nudge**: **FS/MF:** between scapular spines | 0 1 2 3 4 | 0 1 2 3 4 | NA |
| **Lateral Nudge**:  **FS**: L/R Acromion, *use lower score*  **FM**: Dominant side acromion | 0 1 2 3 4  Dominant Side: **L R** | **L**: 0 1 2 3 4  **R**: 0 1 2 3 4 | NA |
| 1. **Static Sitting:** eyes open   **MF/FS:** 30s  **TCT**: 10s | 0 1 2 3 4 | 0 1 2 3 4 | 0: Falls  1: Need support of upper limbs  2: Maintains position for 10 sec |
| **NUDGE AND REPORT ABOVE NUDGE AND REPORT ABOVE NUDGE AND REPORT ABOVE** | | | |
| 2. **Static Sitting**: M**F/FS**: eyes closed- 30 s | 0 1 2 3 4 | 0 1 2 3 4 | NA |
| 3. **Sitting Head Rotation**: L/R   **MF**: Hold 2 sec each side  **FS:** Hold 3 seconds each side | 0 1 2 3 4 | 0 1 2 3 4 | NA |
| 4. **Sitting, lift thigh/unweight foot:**   **MF:** lift 1 inch for 3 seconds **/ FS:** unweight | 0 1 2 3 4 | 0 1 2 3 4 | NA |
| 5. **Cross-leg Sitting: TCT**: cross one leg over the other, score both sides   *****Score only the ability to maintain position with altered base of support  *****If can’t reach position, make a note in the comments section and circle (NT) | NA | NA | **L**: 0: Falls (NT)  1: Need support of upper limbs  2: Maintains position for 10 sec |
|  |  |  | **R**: 0: Falls (NT)  1: Need support of upper limbs  2: Maintains position for 10 sec |
| 6. **Touch Posterior Tape/item/pickup bottle:**   **MF**: bottle (hand’s breadth posterior to sacrum)  **FS**: tape/item (13cm posterior to hips in midline) | Reaching arm: L R | | NA |
|  | 0 1 2 3 4 | 0 1 2 3 4 |  |
| **NUDGE AND REPORT ABOVE NUDGE AND REPORT ABOVE NUDGE AND REPORT ABOVE** | | | |
| 7. **Forward Lean/Reach:** a) **MM**: **Reach** with dominant arm, movement through full ROM or abdomen contacts thighs, return upright (if arm cannot be lifted, use acromion as point of measure) b) **FS**: **Lean** to unweight ischia, return upright c) **TCT**: **Reach** with GH joint at 90°, arm extended, (or most distal articulation maintained in alignment with the AC joint- can be acromion itself) target in midline, 10cm from fingers- *reach to touch*, L/R | 0 1 2 3 4 | 0 1 2 3 4 | **L:** 0: Not done  1: Requires support of the contralateral limb  2: Done without support |
|  |  |  | **R**: 0: Not done  1: Requires support of the contralateral limb  2: Done without support |
| 8. **Lateral Lean/Reach**: a) **MF**: **Reach** with dominant arm, maintain upright trunk, abducted upper extremity, contralateral trunk shortening, clearance of contralateral ischial tuberosity, return to sit. b) **FS:** **Lean** to L/R to unweight contralateral ischial tuberosity and return upright, *take lower score* c) **TCT**: Place target 45° away from midline, 10 cm from outstretched arm (GH 90 or most distal articulation), *reach to touch*, L/R | 0 1 2 3 4  Dominant Side: **L R** | **L**: 0 1 2 3 4  **R**: 0 1 2 3 4 | **L:** 0: Not done  1: Requires support of the contralateral limb  2: Done without support |
|  |  |  | **R**: 0: Not done  1: Requires support of the contralateral limb  2: Done without support |
| 9. **Cross-Body Reach: TCT**   Keep target 45° away from midline and *reach to touch* (do not grade return) with outstretched contralateral arm (GH 90 or the most distal articulation that can be maintained in alignment with the AC joint- can be acromion itself), L/R | NA | NA | **L**: 0: Not done  1: Requires support of the contralateral limb  2: Done without support |
|  |  |  | **R**: 0: Not done  1: Requires support of the contralateral limb  2: Done without support |
| **NUDGE AND REPORT ABOVE NUDGE AND REPORT ABOVE NUDGE AND REPORT ABOVE** | | | |
| 10. **Touch feet/floor:** a) **MF**: Pick up/touch object between feet b) **FS**: Touch dorsum of foot c) **TCT**: touch feet with **both hands** | 0 1 2 3 4 | 0 1 2 3 4 | 0: Not done  1: Requires support with an upper limb  2: Touches feet with **both hands** |
| 11. **Scooting**: move in **Anterior/Posterior/Lateral** directions 5cm   **MF**: can choose dominant side  **FS**: both L/R, *take lower score* | **Ant**: 0 1 2 3 4 | **Ant**: 0 1 2 3 4 | NA |
|  | **Post**: 0 1 2 3 4 | **Post**: 0 1 2 3 4 |  |
|  | **Lat**: 0 1 2 3 4  Dominant Side: **L R** | **L**: 0 1 2 3 4  **R**: 0 1 2 3 4 |  |
| **NUDGE AND REPORT ABOVE NUDGE AND REPORT ABOVE NUDGE AND REPORT ABOVE** | | | |
| 12. Upright > Supine (legs remain bent over edge of plinth) > Upright: **TCT**   *arm momentum is not aid from upper limbs  *do not allow leg locking under plinth | NA | NA | 0: Not done  1: Requires aid of upper limbs  2: Done without aid |
| 13. Roll onto side (from fully supine (legs on plinth) to prone): **TCT** | NA | NA | **L**: 0: Not done  1: Done (No assist) |
|  |  |  | **R**: 0: Not done  1: Done (No assist) |
| **Total** |  |  |  |
| General Comments per scale>>>  Please note if the patient reported pain or presented with limitations in ROM | If (1) is scored, please document assistance level here with item number. |  |  |
| **ID#: ABS**_______ **Rater: ­­­**_____ **Date:** _______________ | | | |
